# Leucine Supplementation Improves Acquired Growth Hormone Resistance in Rats with Protein-Energy Malnutrition

**DOI:** 10.1371/journal.pone.0125023

**Published:** 2015-04-24

**Authors:** Xuejin Gao, Feng Tian, Xinying Wang, Jie Zhao, Xiao Wan, Li Zhang, Chao Wu, Ning Li, Jieshou Li

**Affiliations:** 1 Research Institute of General Surgery, Jinling Hospital, Southern Medical University, Guangzhou, Guangdong Province, China; 2 Research Institute of General Surgery, Jinling Hospital, Medical School of Nanjing University, Nanjing, Jiangsu Province, China; 3 Department of Neurosurgery, Jinling Hospital, Medical School of Nanjing University, Nanjing, Jiangsu Province, China; University of Cordoba, SPAIN

## Abstract

**Background:**

Protein-energy malnutrition (PEM) can lead to growth hormone (GH) resistance. Leucine supplementation diets have been shown to increase protein synthesis in muscles. Our study aimed at investigating if long-term leucine supplementation could modulate GH-insulin-like growth factor (IGF)-1 system function and mammalian target of rapamycin (mTOR)-related signal transduction in skeletal muscles in a rat model of severe malnutrition.

**Methodology/Principal Findings:**

Male Sprague-Dawley rats (n = 50; weight, 302 ± 5 g) were divided into 5 treatment groups, including 2 control groups (a normal control group that was fed chow and ad libitum water [CON, n = 10] and a malnourished control group [MC, n = 10] that was fed a 50% chow diet). After undergoing a weight loss stage for 4 weeks, rats received either the chow diet (MC-CON, n = 10), the chow diet supplemented with low-dose leucine (MC-L, n = 10), or the chow diet supplemented with high-dose leucine (MC-H, n = 10) for 2 weeks. The muscle masses of the gastrocnemius, soleus, and extensor digitorum longus were significantly reduced in the MC group. Re-feeding increased muscle mass, especially in the MC-L and MC-H groups. In the MC group, serum IGF-1, IGF-binding protein (IGFBP)-3, and hepatic growth hormone receptor (GHR) levels were significantly decreased and phosphorylation of the downstream anabolic signaling effectors protein kinase B (Akt), mTOR, and ribosomal protein S6 kinase 1 (S6K1) were significantly lower than in other groups. However, serum IGF-1 and IGF binding protein (IGFBP)-3 concentrations and hepatic growth hormone receptor (GHR) levels were significantly higher in the MC-L and MC-H groups than in the MC-CON group, and serum IGFBP-1 levels was significantly reduced in the MC-L and MC-H groups. These changes were consistent with those observed for hepatic mRNA expression levels. Phosphorylation of the downstream anabolic signaling effectors Akt, mTOR, and S6K1 were also significantly higher in the MC-L and MC-H groups than in the MC-CON group.

**Conclusion/Significance:**

Our data are the first to demonstrate that long-term supplementation with leucine improved acquired growth hormone resistance in rats with protein-energy malnutrition. Leucine might promote skeletal muscle protein synthesis by regulating downstream anabolic signaling transduction.

## Introduction

Protein-energy malnutrition (PEM) has long been recognized as a common problem among children in developing countries, hospitalized (and especially elderly hospitalized) patients, and cancer patients [1–3]. Several reports have shown that PEM results in an impaired response to therapeutic planning [4], inefficient and delayed wound healing, increased wound bursting strength related to sutures, immune system dysfunction [5,6], increased frequency of infectious and noninfectious complications [7], prolonged hospitalization duration, and increased mortality rates [8,9]. In patients with severe protein restriction (kwashiorkor) or protein-energy deprivation, growth retardation is associated with low serum insulin-like growth factor (IGF)-1 levels despite elevated or normal serum growth hormone (GH) concentrations [10].

IGF-1 is a member of the insulin super-family [11]. It was originally discovered as a mediator of GH action on somatic cell growth but it has also been shown to be an important regulator of cell metabolism, differentiation, and survival. IGF-1 is mainly synthesized in the liver. It is found in blood and other body fluids as a complex with specific high-affinity IGF-binding proteins (IGFBP-1 to IGFBP-6) [12,13]. Nutritional status is a key regulator of circulating and tissue-bound IGF-1 [14]. Protein and energy content of the diet influences plasma IGF-1 concentrations [15], and IGF-1 levels are reduced during conditions of energy restriction such as short-term fasting [16] and malnutrition [17]. Furthermore, it was shown that IGF-1 promotes protein synthesis within the body [18].

IGF-1 synthesis is a well-regulated process that depends on the availability of energy and amino acids. Leucine activates the phosphoinositide-3 kinase (PI3K) anabolic pathway, which in turn triggers a cascade of intracellular signals that culminate in the activation of protein translation. Our previous study found that leucine-enriched high-protein enteral nutrition could promote IGF-1 system synthesis in a rat model of trauma-hemorrhagic shock [19]. Although some studies have demonstrated that intravenous infusion or ingestion of leucine can acutely increase the protein synthesis rate [20,21], only few studies have tested whether long-term supplementation with leucine promotes significant changes in the GH-IGF-1 system during severe malnutrition.

Therefore, the purpose of this study was to investigate if long-term dietary supplementation with different doses of leucine could regulate GH-IGF-1 system function and mammalian target of rapamycin (mTOR)-related signal transduction in skeletal muscles in a rat model of severe malnutrition.

## Materials and Methods

### Animals

Male Sprague-Dawley rats with an initial weight of 302 ± 5 g were provided by the Medical Experiment Animal Center of the Jinling Hospital, Nanjing, China. Animals were housed in individual cages in a climatized environment at 23 ± 2°C and a relative air humidity of 55 ± 10% under a 12-h light/12-h dark cycle (lights were on from 8:00 A.M. to 8:00 P.M.) for 8 weeks. Animals had free access to water throughout the experiment. All animal experiment protocols and procedures were approved by the Animal Care and Use Committee of the Jinling Hospital (No201346) and complied with the principles of laboratory animal care (NIH publication No. 86–23, revised 1985).

### Diet and Study Design

The chow diet was prepared according to the 1993 recommendations of the American Institute of Nutrition for adult rats (AIN-93M). Initially, animals were fed the AIN-93M diet *ad libitum* for 2 weeks to let them adapt to the new environmental conditions and to determine their food intake. The rats’ mean food intake was used to determine the amount of chow offered daily during the food restriction phase.

Animals were randomized into 5 groups: the control group (CON, n = 10) receiving a chow diet for 6 weeks and *ad libitum* water, the malnourished control group (MC, n = 10) subjected to 50% food restriction for 4 weeks, the MC-CON group (subjected to 50% food restriction for 4 weeks, followed by 2 weeks of receiving a chow diet, n = 10), the MC-L group (subjected to 50% food restriction for 4 weeks, followed by 2 weeks of receiving a chow diet supplemented with low-dose leucine [0. 675 g leucine/kg of body weight] by oral gavage, n = 10), and the MC-H group (subjected to 50% food restriction for 4 weeks, followed by 2 weeks of receiving a chow diet supplemented with high-dose leucine [1.35 g leucine/kg of body weight] by oral gavage, n = 10).(see S1 Fig)

### Sample Collection

All animals were sacrificed by anesthesia with ketamine (100 mg/kg, i.p.), and blood (about 3 ml) was immediately collected in a dry tube. Blood samples were allowed to clot for 2 h at room temperature and were then centrifuged for 20 min at 2,000 × *g*. Serum was removed, and the samples were stored at -80°C until further analysis. After blood collection, a part of the liver and the entire gastrocnemius, soleus, and extensor digitorum longus muscles were collected, weighed, wrapped, and immersed in liquid nitrogen. This process lasted no longer than 3 min after the death of the animal. All samples were stored frozen at -80°C until further analysis.

### Body and Skeletal Muscle Wet Weight

Changes in body weight and rat skeletal muscle wet weight were assessed by electronic balance.

### Measurement of Serum GH, IGF-1, IGFBP-1, and IGFBP-3 Levels by Enzyme-linked Immunosorbent Assay (ELISA)

Serum concentrations of GH, IGF-1, IGFBP-1, and IGFBP-3 were determined by ELISA kits in accordance with the manufacturer’s instructions (GH: Millipore, MN, USA; IGF-1, IGFBP-1, and IGFBP-3: R&D, MN, USA).

### Measurement of Hepatic Tissue mRNA for GHR, IGF-1, and IGFBPs

The primers used for real-time quantitative reverse transcription (RT)-PCR are depicted in S1 Table. Total RNA was extracted from the liver using the PrimeScript RT reagent Kit with cDNA (TaKaRa, MN, Japan) according to the manufacturer’s instructions. Total RNA (1 μg) was treated with DNase I and used for the RT reaction. The synthesized cDNA was stored at -20°C until further use. Real-time quantitative (q) RT-PCR was performed with the iCycler iQ system (ABI, MN, USA) and the SYBR Green I fluorescence kit (Sigma, MN, USA) according to the manufacturer's instructions. Amplification of mRNAs was performed in duplicate in a 96-well PCR reaction plate (ABI, MN, USA). The following real-time qRT-PCR protocol was used: cDNA was denatured at 95°C for 5 min to activate the Hot-start Taq DNA polymerase. The amplification and quantification program was repeated 40 times (95°C for 30 s, 95°C for 5 s, and 65°C for 31 s).The mRNA expression of GHR, IGF-1, IGFBP-1 and IGFBP-3 was measured using the 2^-△△CT^ method.

### Western Blot Analysis

Muscle and liver preparations (0.1 g frozen muscle and liver samples) were homogenized in 1 mL RIPA lysis buffer (25 mM Tris-HCl pH 7.6, 150 mM NaCl, 1% NP-40, 1% sodium deoxycholate, and 0.1% sodium dodecyl sulfate [SDS]) containing 200 mM NaF, 1 mM Na_3_VO_4_, 25 mM β-glycerophosphate, 1 mM PMSF, and 1% protein inhibitor cocktail) for 90 min at 4°C in a cold room while lightly shaking with constant speed. The lysate was then centrifuged twice at 12,000 × *g* at 4°C for 10 min, and the supernatant was collected. Total protein concentration was determined using the BCA assay. (Sangon Biotech Co., Shanghai, China). The supernatant was diluted (4:1) in a 4× loading buffer and then boiled at 100°C for 5 min. Equal amounts and volumes of protein (64 μg total protein) were separated by 8% or 10% SDS-polyacrylamide gel electrophoresis based on the size of the target protein and then transferred to a polyvinylidene fluoride membrane (Millipore Co, Billerica, MA, USA). Blots were blocked in 5% non-fat milk for 1 h at room temperature and incubated in specific primary antibodies overnight at 4°C in a cold room while shaking. Blots were washed 3 times in Tris-buffered saline with Tween and incubated with the secondary antibody for 1 h at room temperature. Protein bands were visualized by a chemiluminescence detection system, and the blots were exposed to a Kodak XAR film (Eastman Kodak, MN,USA). Band density was analyzed using the ImageJ software. Specific total protein was reprobed after stripping the phospho-primary and secondary antibodies. Phosphorylation data are described relative to total protein expression after normalization by the internal loading control.

### Antibodies

Antibodies for GAPDH (1:10,000), mTOR (1:1,000), phospho-mTOR (Ser2448; 1:500), S6 kinase 1 (S6K1; 1:1,000), phospho-S6K1 (Thr389; 1:500), protein kinase B (PKB or Akt; 1:1,000), and phospho-Akt (Ser473; 1:1,000) were purchased from Cell Signaling Technology (Beverly, MA, USA).The GHR (1:1,000) antibody was obtained from Abcam (Cambridge, MA, UK), and the anti-rabbit IgG horseradish peroxidase-conjugated secondary antibody (1:5,000) from Cell Signaling Technology (Beverly, MA, USA).

### Statistical Analysis

Data are expressed as means ± standard deviation (SD). Statistical evaluation among groups was performed by one-way ANOVA followed by the Fisher’s least significant difference post-hoc analysis, using the SPSS19.0 software (SPPS Inc. Chicago, IL, USA). In the case of heterogeneous variances, we used the Dunnet’s T3 test. A p value of <0.05 was considered statistically significant.

## Results

### Changes in Body and Skeletal Muscle Wet Weight

**Fig 1:**
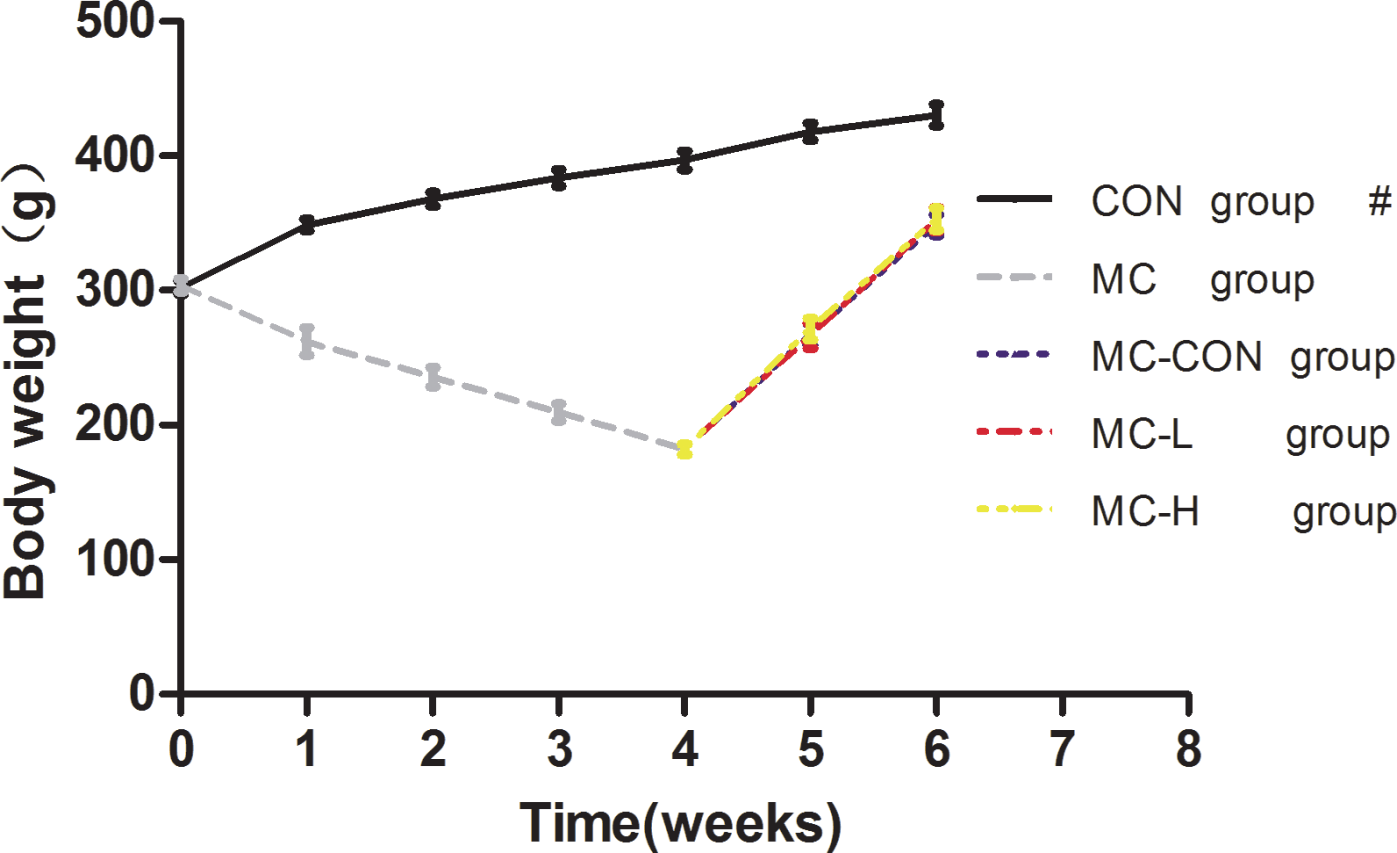
Body Weight of the Rats. Values are presented as means ± standard deviation (SD). # indicates a significant difference, p <0.01.

Rats weighed an average of 302 ± 5 g at the beginning of our study. Body weight was low in all MC rats throughout the study, but did increase during the re-feeding period (p < 0.01, Fig 1). Re-feeding (and in particular, leucine supplementation) was beneficial for achieving weight gain; however, no significant differences between the groups were observed.

**Fig 2:**
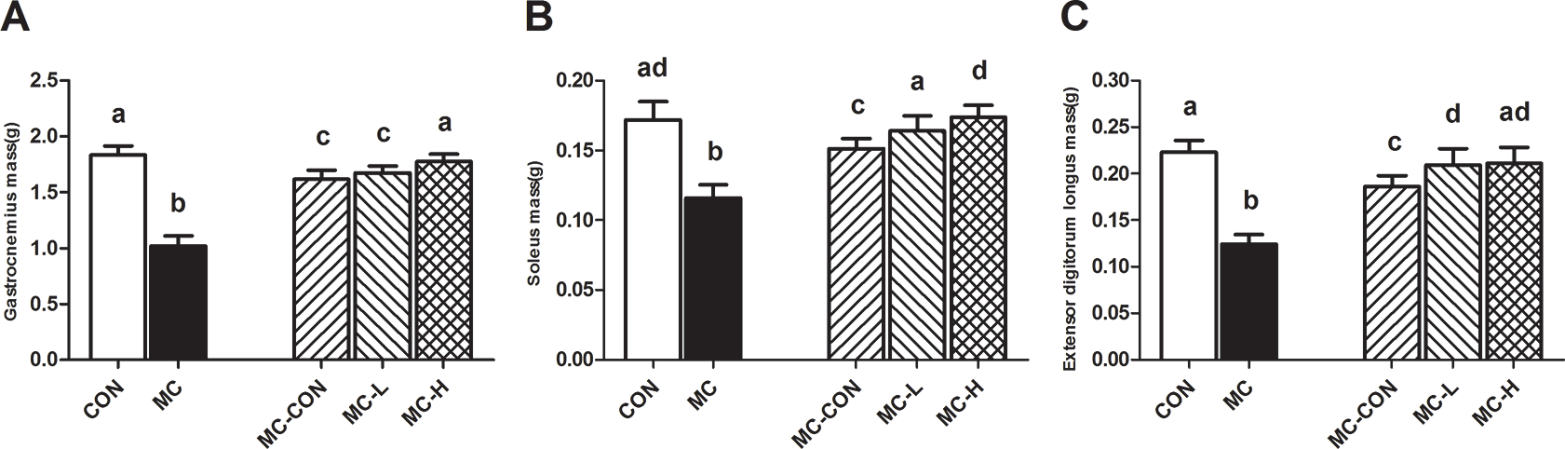
Comparison of Skeletal Muscle Masses. Wet masses of the gastrocnemius (A), soleus (B), and extensor digitorum longus (C) were measured for each group at the study end point. Values are presented as means ± standard deviation (SD). Different letters indicate a significant difference (p <0.05).

Significant reductions in wet weight caused by malnutrition were found in the gastrocnemius, soleus, and extensor digitorum longus (Fig 2). The wet weights of the gastrocnemius, soleus, and extensor digitorum longus muscles in the MC-L and MC-H groups were significantly higher than those in the MC-CON group at the study end point (i.e., after re-feeding) (p < 0.05, Fig 2). However, no significant differences were observed between the MC-L, MC-H, and CON groups.

### Serum GH, IGF-1, IGFBP-1, and IGFBP-3 concentrations

**Fig 3:**
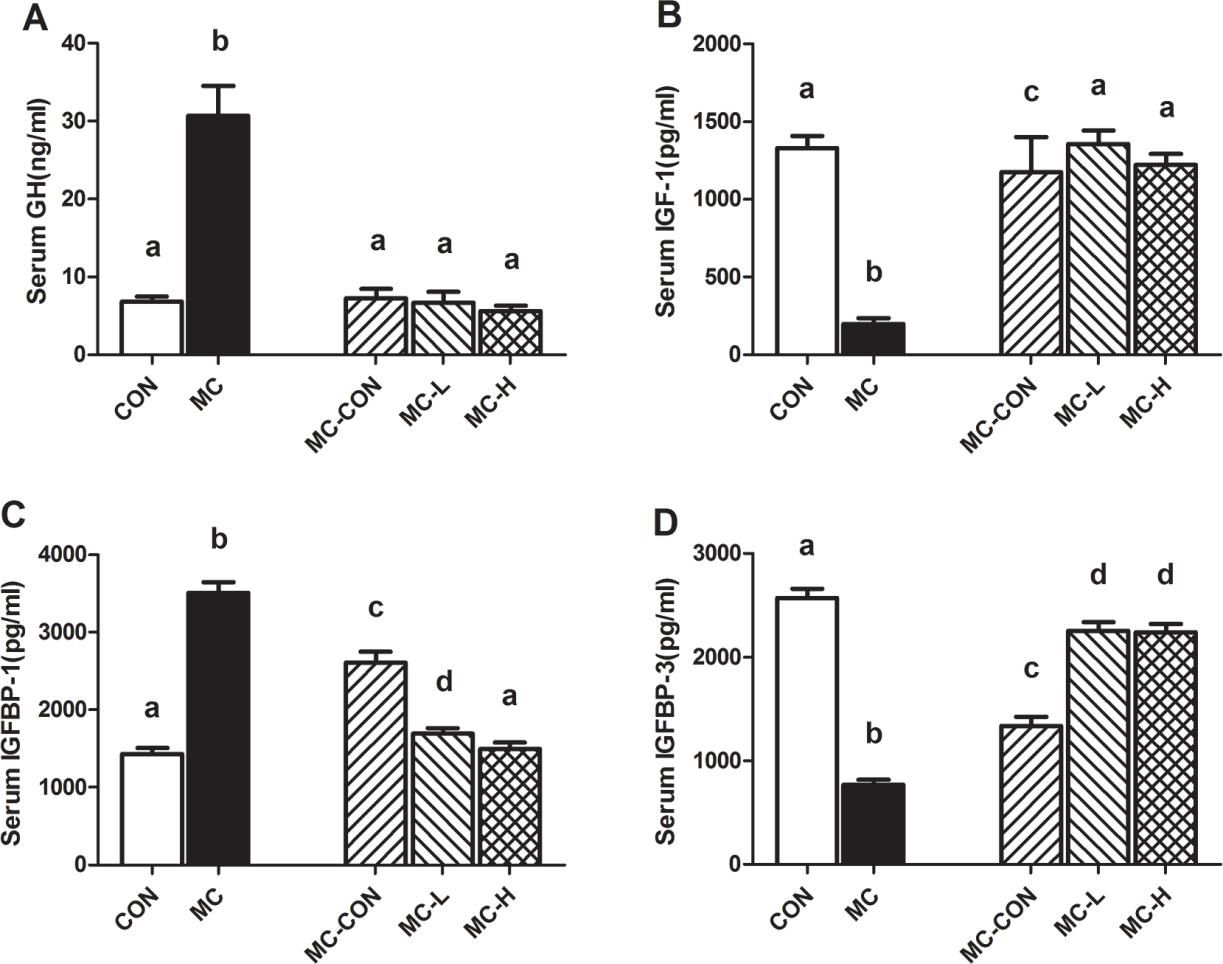
Serum Levels of GH, IGF-1, IGFBP-1, and IGFBP-3. GH (A), IGF-1 (B), IGFBP-1 (C), and IGFBP-3 (D) levels in each group were measured by enzyme-linked immunosorbent assay (ELISA). Values are presented as means ± standard deviation (SD). Different letters indicate a significant difference (p <0.05). GH, growth hormone; IGF-1, insulin-like growth factor-1, IGFBP-1/-3, insulin-like growth factor binding protein-1/-3.

At the study end point (after re-feeding), GH levels were significantly higher in the MC than the CON group (p < 0.01, Fig 3A). However, no statistical differences in GH levels were noted between the MC-CON, MC-L, MC-H, and CON groups (p > 0.05, Fig 3A).

Serum IGF-1 levels were significantly lower in the MC and MC-CON groups compared to those of the CON group (p < 0.01 and p < 0.05, respectively, Fig 3B). Moreover, serum IGF-1 levels in the MC-L and MC-H groups were significantly higher than in the MC-CON group (p < 0.01, Fig 3B). No differences in IGF-1 levels were observed between the MC-L, MC-H, and CON groups.

Serum IGFBP-1 levels in the CON group were markedly lower than in the other groups at the study end point. The MC-CON group showed higher IGFBP-1 concentrations than the MC-L and MC-H groups (p < 0.05, Fig 3C). Serum IGFBP-1 levels in the MC group were significantly higher than those in the other groups (p < 0.01, Fig 3C). However, we found no difference in IGFBP-1 levels between the MC-H and CON groups.

Serum IGFBP-3 levels among the groups were similar to those observed for IGF-1. In the MC group, IGFBP-3 levels remained lower than in the other groups at the study end point (p < 0.01, Fig 3D). Following re-feeding, we detected significantly elevated IGFBP-3 levels in all groups, when compared with the MC group (p < 0.01, Fig 3D). IGFBP-3 levels in the MC-L and MC-H groups were 168% and 167%, respectively, higher than those in the MC-CON group at the end of the study (p < 0.01, Fig 3D)

### Hepatic expression of GHR, IGF-1, IGFBP-1, and IGFBP-3 mRNA

**Fig 4:**
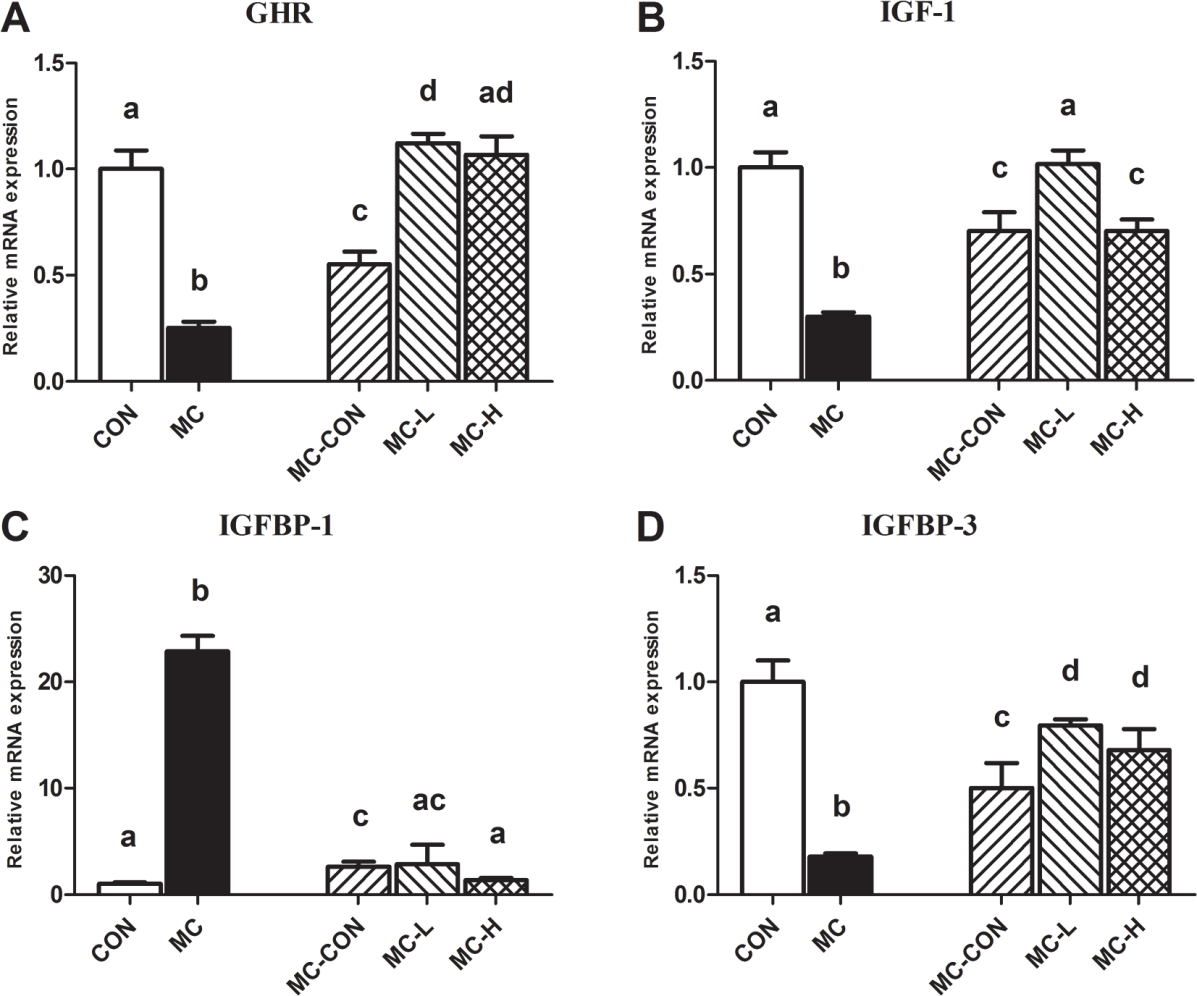
Hepatic Expression of GHR, IGF-1, IGFBP-1, and IGFBP-3 mRNA. GHR (A), IGF-1 (B), IGFBP-1 (C), and IGFBP-3 (D) mRNA levels in each group were measured by real-time quantitative reverse transcriptase (RT)-PCR. Values are presented as means ± standard deviation (SD). Different letters indicate a significant difference (p <0.05). GHR, growth hormone receptor, IGF-1, insulin-like growth factor-1, IGFBP-1/-3, insulin-like growth factor binding protein-1/-3.

The relative expression of GHR mRNA was significantly higher in MC-L rats than in CON, MC, MC-CON, and MC-H rats (p < 0.045, p < 0.000, p < 0.000, and p = 0.338, respectively; Fig 4A). We found the lowest expression levels for GHR in the MC group (CON, 100%; MC, 25.2%; MC-CON, 55.2%; MC-L, 112.2%; and MC-H, 106.8%; Fig 4A).

The relative expression of IGF-1 mRNA was lower in the MC and MC-CON groups than the CON and MC-L groups (p < 0.000, Fig 4B). We found no statistically significant difference in IGF-1 mRNA levels among the other groups.

IGFBP-1 mRNA levels were significantly higher in the MC than the CON group. However, levels in the MC group were significantly higher than in the other groups (p < 0.000, Fig 4C).

After re-feeding, the relative expression of IGFBP-3 mRNA in the MC group was lower than that in all other groups. We observed significant differences in IGFBP-3 mRNA levels among the MC-L, MC-H, and MC groups, but no difference was found between the MC-L and MC-H groups.

### Cell Signaling

To further assess signal transduction associated with GHR synthesis in muscle and liver tissue, a number of phosphorylation key points of proteins involved in the mTOR signaling pathway and the expression of GHR in hepatic tissue were determined at the end point of the study.

**Fig 5:**
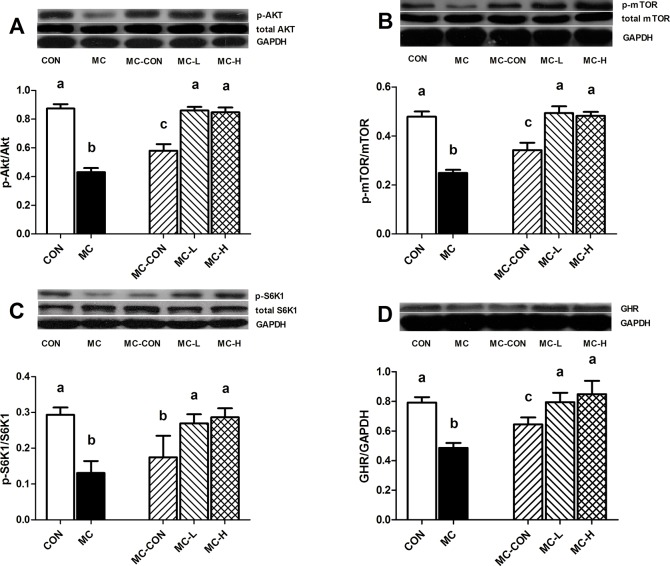
Phosphorylation of mTOR-related Signaling Effectors in the Gastrocnemius. Akt phosphorylation at Ser473 and total Akt (A), mTOR phosphorylation at Ser2448 and total mTOR (B), p70-S6K1 phosphorylation at Thr389 and total p70-S6K1 (C), and GHR expression levels in the liver (D) were assessed by western blot. The band densities were quantified by the ImageJ software. Data are presented as means ± standard deviation (SD). Different letters indicate a significant difference (p < 0.05). Akt, protein kinase B; GHR, growth hormone receptor; mTOR, mammalian target of rapamycin; p70-S6K1, ribosomal protein S6 kinase 1.

Phosphorylation of Akt at Ser473 in the MC-CON group was greatly suppressed in comparison to the CON, MC-L, and MC-H groups (p < 0.01, p < 0.01, and p < 0.01, respectively; Fig 5A). Moreover, phospho-Akt expression levels in the MC group were significantly lower than that in the MC-CON, MC-L, MC-H, and CON groups (p < 0.01, p < 0.01, and p < 0.01, respectively; Fig 5A).

Phosphorylation levels of mTOR at Ser2448 in the MC group were also lower than in the other groups (p < 0.01, Fig 5B). We found higher mTOR phosphorylation in the MC-L and MC-H groups than in the MC-CON group (p < 0.01 and p < 0.01, respectively; Fig 5B).

Phosphorylation of S6K1 (an important downstream effect of mTOR signaling) was similar in the MC-L and MC-H groups, but markedly higher in these two groups than in the MC and MC-CON groups (p < 0.01 and p < 0.01, respectively; Fig 5C). No statistically significant difference in S6K1 phosphorylation was observed between the MC-L and MC-H groups and the CON group (p > 0.05, Fig 5C).

GHR levels in hepatic tissue were higher in the MC-L and MC-H groups than the MC and MC-CON groups (p < 0.05, Fig 5D). However, no statistically significant difference in GHR levels was observed among the MC-L, MC-H, and CON groups.

## Discussion

In our study, the significantly reduced skeletal muscle masses caused by malnutrition were increased during re-feeding, especially in rats receiving leucine supplementation. Moreover, serum IGF-1, IGFBP-3, and hepatic GHR levels were significantly higher in the MC-L and MC-H groups compared to the MC-CON group at the study end point. We also showed that phosphorylation levels of the signaling effectors Akt, mTOR, and S6K1 were significantly higher in the MC-L and MC-H groups. Our data thus indicate that long-term leucine supplementation improved acquired growth hormone resistance in rats with PEM.

PEM is classified as slight (10–25%), moderate (25–39%), or severe (>40%) based on the amount of weight loss [22]. Our study used a 50% food restriction, resulting in severe malnutrition in our animal model. After 4 weeks, the MC animals lost 40% of their body weight, which equals severe malnutrition, indicating that our experimental model was successfully established.

Low IGF-1 levels were associated with increased serum GH levels in the MC group. Similar data have been previously reported by others [23,24]. Patients suffering from malnutrition with GH hypersecretion and low IGF-1 levels are considered GH-resistant. This hormonal pattern is characteristic of a catabolic state [25]. Our study revealed that leucine supplementation resulted in the rapid recovery of the IGF-1 system in rats with severe malnutrition. We also observed muscle mass maintenance, downstream mTOR pathway activation, and GHR synthesis, indicating effective stimulation of muscle and liver protein synthesis. These results provide valuable insight into how long-term supplementation with only leucine can improve GH resistance in patients with malnutrition.

Our study clearly demonstrated that serum IGF-1 and IGFBP-3 levels were dramatically lower in rats with malnutrition. The plasma concentration of IGF-1, which is primarily determined by hepatic synthesis and secretion of the peptide hormone, is also dramatically decreased during protein and energy restriction diets. Numerous clinical and animal experiments have shown that PEM not only decreases the synthesis rate of IGF-1 but also enhances its serum clearance and degradation [10]. IGFBP-3 binds the majority of circulating IGFs together with an acid labile protein subunit (ALS) and forms a stable ternary complex that prolongs IGF half-life, functions as a reservoir for IGFs, and limits their extravascular transit.

Reduced hepatic IGF-1 and IGFBP-3 mRNA expression and serum protein levels observed in the rats with malnutrition in our study confirm the findings of previous studies [10,26]. Our study indicated that food deprivation resulted in significantly increased hepatic IGFBP-1 mRNA and serum protein levels. A number of studies have examined the effects of caloric and/or protein restriction on IGF bioactivity. Jackson Smith and colleagues showed that energy restriction decreased IGFBP-3 levels in children but not adults, and that protein restriction decreased IGFBP-3 levels in adults only [27]. In an animal study, Oster et al. noted a significant decrease in IGFBP-3 levels only in rats with a caloric intake that was restricted to 40% of that of controls or those that experienced severe protein restriction [28]. Data from a Northern hybridization analysis showed that IGFBP-3 mRNA levels declined by 30% in rats with protein restriction, and that serum IGFBP-3 levels paralleled this decrease, suggesting transcriptional regulation [29]. In another study by Jackson Smith et al., a rapid increase in IGFBP-1 levels was observed in calorically restricted adults. This increase was reversed with re-feeding [27]. This clinical finding contrasts data from a study by Straus et al. who demonstrated that prolonged protein restriction in a rat model caused a greater induction of IGFBP-1 gene expression than did short-term fasting [30]. These authors also demonstrated that amino acid restriction increased the abundance of IGFBP-1 mRNA in rat hepatoma cells, stressing the importance of both insulin and protein substrate in the regulation of IGFBP-1. These findings suggest that IGFBP-1 may function to decrease IGF bioactivity during low nutritional substrate availability.

Previous studies using leucine-enriched formula or leucine alone to investigate the stimulatory effect of these compounds on skeletal muscle protein synthesis did not demonstrate changes in serum IGF-1 and IGFBP levels. Meanwhile, acute dosing of leucine solution alone during inadequate nutrient availability is not sufficient for stimulation of IGF-1 production. Our study indicates that re-feeding with a leucine supplementation diet (in the MC-L and MC-H groups) resulted in significantly higher serum IGF-1 and IGFBP-3 levels than those found in MC-CON rats (that did not receive leucine). This is a desirable outcome, since IGF-1 plays an important role in GH resistance during malnutrition. We also found that IGF-1 and IGFBP-3 mRNA levels significantly increased in the MC-L and MC-H groups. Previous studies have found that re-feeding with a diet consisting of essential amino acids (e.g., leucine) resulted in a larger increase in serum IGF-1 levels than re-feeding with non-essential amino acids, further supporting our findings [31].

Our data show that hepatic GHR and GHR mRNA levels were clearly lower in the MC group than in the CON group, providing one explanation for decreased serum and hepatic IGF-1 mRNA levels. Straus and Takemoto showed that GHR mRNA abundance in the liver declined during fasting and responded to re-feeding [32]. In our study, hepatic GHR and GHR mRNA levels after re-feeding were significantly higher in the MC-L and MC-H groups than the MC group, but not significantly different to the CON group. The PI3K-Akt pathway, which acts upstream of mTOR, can be phosphorylated by the ligand IGF-1, which in turn stimulates mTOR phosphorylation to induce liver protein synthesis. Previous studies demonstrated the rapid development of GH resistance along with a decrease in GHR during malnutrition [26]. Therefore, the diminished signal transduction associated with the PI3K-Akt pathway may be partially responsible for GH resistance during severe malnutrition. Our study indicates that leucine plays an important role in GH resistance in rats with severe malnutrition.

After re-feeding with the chow diet for 2 weeks, we found that rats with severe malnutrition recovered weight up to levels that were close to those of the CON group. In addition, our results show that leucine supplementation caused significant improvement in some indicators of protein nutritional status, as observed by the increase in skeletal muscle protein masses. It was not surprising to us that the wet weight of skeletal muscle proteins in the MC-L and MC-H groups did not exhibit a difference to those of the control group, since some studies provided evidence indicating that an increase in leucine intake may help protein loss [21,33]. We also showed that leucine supplementation significantly enhanced the activation of the mTOR signaling pathway. Phosphorylation of mTOR and its downstream effector S6K1 in rats receiving leucine significantly increased compared with rats receiving the chow diet. Recently, a large body of evidence has established that amino acids, especially leucine, can be considered as signaling molecules stimulating protein translation through an Akt-independent pathway. Acute leucine ingestion can effectively increase protein synthesis and promote mTOR-related signal transduction [21,33]. Previous studies demonstrated that IGF-1 administration effectively attenuates the inhibition of protein synthesis during malnutrition and ameliorates the loss of muscle mass. Therefore, in addition to the nutrient-dependent mechanism described above, the increased mTOR anabolic signaling in the rats receiving the leucine supplementation diet in our study may have been partially caused by the activation of the IGF-1 signaling pathway.

In conclusion, oral administration of leucine improved GH resistance in rats with malnutrition by promoting IGF-1, reducing IGF-1 degradation, and facilitating GHR synthesis in the liver. Leucine might activate muscle protein synthesis by regulating mTOR anabolic signaling transduction.

## Supporting Information

S1 Fig Study Design. Our study included 5 treatment groups, including 2 control groups (a normal control group that was fed chow and *ad libitum* water [CON] and a malnourished control group [MC] that was fed a 50% chow diet). After undergoing the weight loss stage, rats received either the chow diet (MC-CON), the chow diet supplemented with low-dose leucine (MC-L), or the chow diet supplemented with high-dose leucine (MC-H).
(TIF)

S1 Table Primers Used for Reverse Transcriptase Quantitative PCR Assays. (DOCX)
