# Supplementary figures and images for: Leucine Supplementation Improves Acquired Growth Hormone Resistance in Rats with Protein-Energy Malnutrition
Source: PLoS One. 2015 Apr 24;10(4):e0125023. doi: 10.1371/journal.pone.0125023 (PMC4409315; doi:10.1371/journal.pone.0125023)

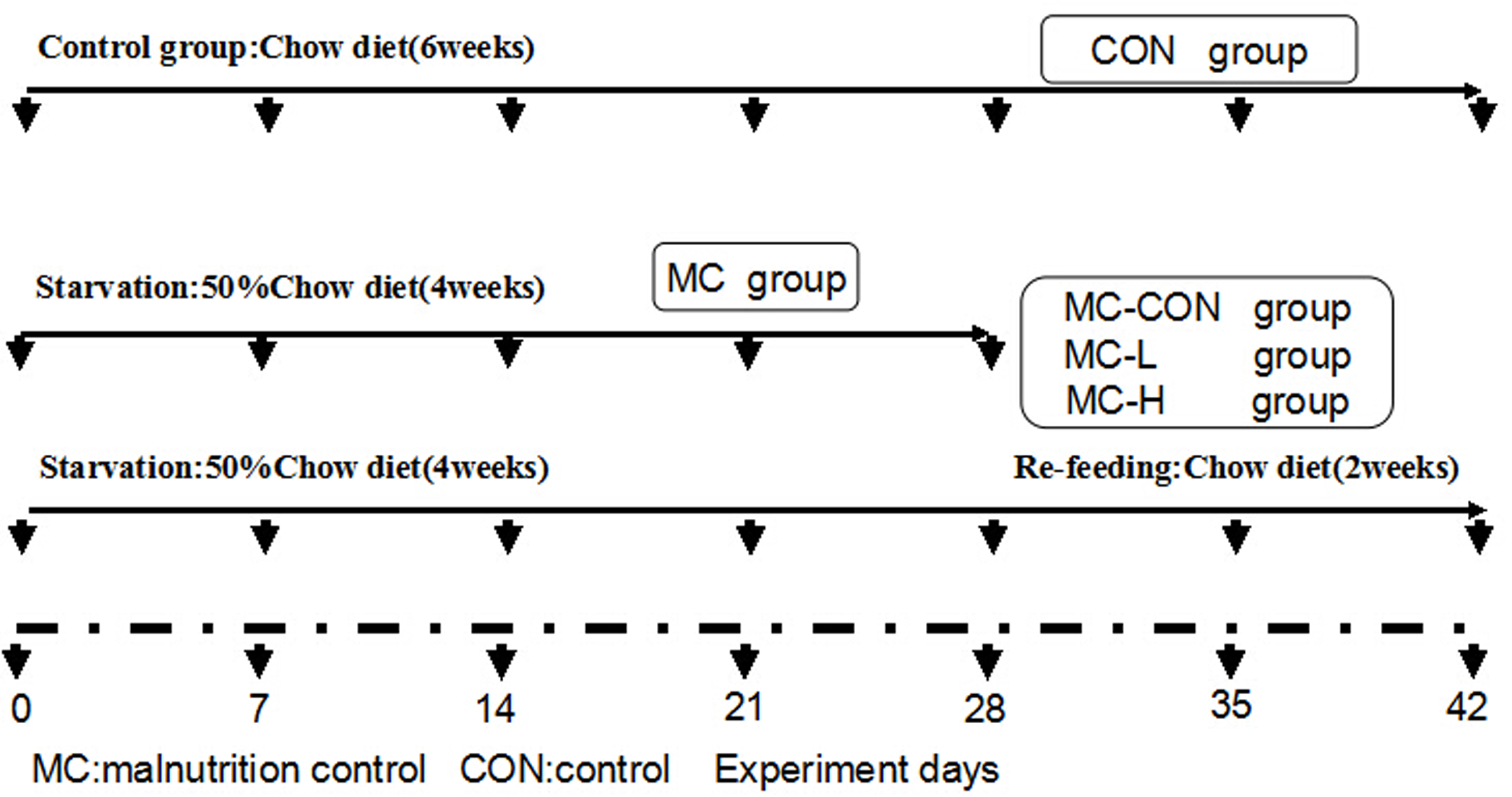

Supplement: S1 Fig — Our study included 5 treatment groups, including 2 control groups (a normal control group that was fed chow and ad libitum water [CON] and a malnourished control group [MC] that was fed a 50% chow diet). After undergoing the weight loss stage, rats received either the chow diet (MC-CON), the chow diet supplemented with low-dose leucine (MC-L), or the chow diet supplemented with high-dose leucine (MC-H). (TIF) [file pone.0125023.s001.tif]
