# Supplementary material for: Leucine Supplementation Improves Acquired Growth Hormone Resistance in Rats with Protein-Energy Malnutrition
Source: PLoS One. 2015 Apr 24;10(4):e0125023. doi: 10.1371/journal.pone.0125023 (PMC4409315; doi:10.1371/journal.pone.0125023)
Supplement: S1 Table — (DOCX) [file pone.0125023.s002.docx]

**S1 Table Primers Used for Reverse Transcriptase Quantitative PCR Assays**.

| **Gene product** | **Forward primer** | **Reverse primer** |
| --- | --- | --- |
| **GHR** | **5'-ATCTTTGGCGGGTGTTCTTA-3'** | **5'-TAGCTGGTGTAGCCCCACTT-3'** |
| **IGF-1** | **5'-TCAGTTCGTGTGTGGACCAAG-3'** | **5'-TCACAGCTCCGGAAGCAAC-3'** |
| **IGFBP-1** | **5′-CTACCCATGGAGTGGGAAGA-3′** | **5′-TGCCCTTTCAAAGCAGAACT-3′** |
| **IGFBP-3** | **5′-AGCCGTCTCCTGGAAACACC-3′** | **5′-CCCGCTTTCTGCCTTTGG-3′** |
| **GAPDH** | **5′- AGGCCGGTGCTGAGTATGTC-3′** | **5′- TGCCTGCTTCACCACCTTCT-3′** |

GAPDH, glyceraldehyde 3-phosphate dehydrogenase; GHR, growth hormone receptor, IGF-1, insulin-like growth factor-1, IGFBP-1/-3, insulin-like growth factor binding protein-1/-3
